# Evaluation of a large language model to simplify discharge summaries and provide cardiological lifestyle recommendations

**DOI:** 10.1038/s43856-025-00927-2

**Published:** 2025-05-29

**Authors:** Paul Rust, Julian Frings, Sven Meister, Leonard Fehring

**Affiliations:** 1 Faculty of Health, School of Medicine, Witten/Herdecke University, Alfred-Herrhausen-Strasse 50, 58455 Witten, Germany; 2 Health Care Informatics, Faculty of Health, School of Medicine, Witten/Herdecke University, Pferdebachstrasse 11, 58455 Witten, Germany; 3 Department Healthcare, Fraunhofer Institute for Software and Systems Engineering ISST, Speicherstrasse 6, 44147 Dortmund, Germany; 4 Helios University Hospital Wuppertal, Department of Gastroenterology, Witten/Herdecke University, Heusnerstrasse 40, 42283 Wuppertal, Germany

**Keywords:** Health care, Cardiology

## Abstract

**Background:**

Hospital discharge summaries are essential for the continuity of care. However, medical jargon, abbreviations, and technical language often make them too complex for patients to understand, and they frequently omit lifestyle recommendations important for self-management. This study explored using a large language model (LLM) to enhance discharge summary readability and augment it with lifestyle recommendations.

**Methods:**

We collected 20 anonymized cardiology discharge summaries. GPT-4o was prompted using full-text and segment-wise approaches to simplify each summary and generate lifestyle recommendations. Readability was measured via three standardized metrics (modified Flesch-Reading-Ease, Vienna Non-fiction Text Formula, Lesbarkeitsindex), and multiple quality dimensions were evaluated by 12 medical experts.

**Results:**

LLM-generated summaries from both prompting approaches are significantly more readable compared to the original summaries across all metrics (*p* < 0.0001). Based on 60 expert ratings for the full-text approach and 60 for the segment-wise approach, experts ‘(strongly) agree’ that LLM-summaries are correct (full-text: 85%; segment-wise: 80%), complete (78%; 92%), harmless (83%; 88%), and comprehensible for patients (88%; 97%). Experts ‘(strongly) agree’ that LLM-generated recommendations are relevant in 92%, evidence-based in 88%, personalized in 70%, complete in 88%, consistent in 93%, and harmless in 88% of 60 ratings.

**Conclusions:**

LLM-generated summaries achieve a 10th-grade readability level and high-quality ratings. While LLM-generated lifestyle recommendations are generally of high quality, personalization is limited. These findings suggest that LLMs could help create more patient-centric discharge summaries. Further research is needed to confirm clinical utility and address quality assurance, regulatory compliance, and clinical integration challenges.

## Plain language summary

Discharge summaries contain important information for patients and their doctors after a hospital stay. However, they are often too complicated for patients to understand and usually lack lifestyle advice for home care. We tested if an artificial intelligence (AI) tool, called a large language model, could simplify existing summaries and add lifestyle recommendations. Using standard readability tests, we found the AI-generated summaries were much easier to understand than the originals. Medical experts then reviewed the AI-summaries and rated them as generally accurate, complete, and safe. The AI-lifestyle recommendations were also rated positively, but they were not always personalized to the individual patient. This suggests that AI could help hospitals communicate better with patients, but more research is needed to ensure its safety.

## Abstract (web summary)

Rust et al. evaluate a large language model to simplify cardiology discharge summaries and provide lifestyle recommendations. Findings show improved readability while maintaining medical accuracy, as well as relevant, but not sufficiently personalized, lifestyle recommendations.

## Introduction

Effective communication between healthcare professionals and patients underpins shared decision-making^1^. Discharge summaries play a pivotal role in this process, providing critical information such as diagnoses, treatment plans, medications, and follow-up recommendations. In Germany, providing discharge summaries to patients is legally required, yet they are primarily designed for provider-to-provider communication^2,3^. As a result, they often contain complex medical jargon, abbreviations, and technical details that can be difficult for patients to comprehend. This issue is particularly challenging for people with limited health literacy (59% of the German population), who may struggle to fully understand their own health conditions and treatment plans^4–6^.

Research indicates that patient-centered discharge summaries can improve patients’ health literacy, patient satisfaction, and understanding^7–10^. Despite these benefits, such summaries are virtually nonexistent in clinical practice. Physicians also favor creating separate summaries for patients and fellow providers; however, they often do not have the time and resources to do so^11,12^.

Large Language Models (LLMs), including OpenAI’s Chat Generative Pre-trained Transformer (ChatGPT), offer a promising avenue to automatically transform existing discharge summaries into more patient-centered versions. These models can be tasked to ‘simplify’ discharge summaries by translating specialized medical terminology into plain language, explaining complex concepts, and reorganizing information to improve readability for lay audiences. Although not specifically trained for text simplification, these models have shown considerable potential in medical fields, such as radiology^13–15^ and dermatology^16^ (for a comprehensive overview, see Busch et al.^17^).

Despite promising preliminary findings, further research is needed on LLMs in medical text simplification, as existing studies are sparse and primarily focus on radiology reports, English-language texts, and stylized vignettes. These vignettes fail to capture the complexities of real-world clinical data, such as uncommon abbreviations or incomplete sentence structures. Another potential enhancement is the inclusion of personalized lifestyle recommendations, important for primary and secondary prevention, as they are often omitted due to time constraints. While LLMs can generate relevant recommendations when prompted, their capacity to automatically produce lifestyle advice directly from discharge summaries has seen limited investigation^18^.

This exploratory study aims to address the research gaps in simplifying non-English, real-world discharge summaries and generating lifestyle recommendations. Specifically, we evaluate LLMs’ potential to create more patient-centered discharge summaries by evaluating their effectiveness in (1) enhancing the readability of standard discharge summaries to a level appropriate for laypeople and (2) augmenting these summaries with personalized lifestyle recommendations.

Our findings suggest that an LLM can substantially improve the readability of discharge summaries, as measured by standardized readability metrics, while largely preserving the quality of information, as assessed by medical experts. Additionally, while the LLM generates a wide range of lifestyle recommendations, these lack personalization.

## Methods

**Fig. 1:**
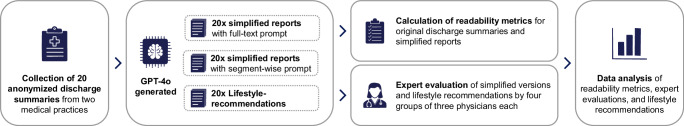
Data collection, processing and analysis workflow.

We conducted a prospective, exploratory study utilizing real-world, anonymized discharge summaries of patients with cardiological conditions. The focus on cardiological diseases was motivated by their high prevalence, as well as the impact of lifestyle modifications on disease management and prevention^19,20^. The summaries were processed through an LLM to simplify the content and add personalized lifestyle recommendations. The outputs were then analyzed for readability using software and assessed for their quality by a panel of medical experts (Fig. 1). Written informed consent was obtained from the participating physicians who provided the anonymized discharge summaries, as well as from the physicians participating in the medical expert panel. The ethics committee of Witten/Herdecke University raised no objection regarding ethical concerns (Number: S-66/2024). The reporting of this study followed the Transparent Reporting of a Multivariable Model for Individual Prognosis Or Diagnosis (TRIPOD) + LLM guideline^21^.

### Collection of the original discharge summaries

We obtained 20 hospital discharge summaries from two German general practitioner offices. These offices received the summaries from various hospitals as part of the discharge transfer process. All summaries were written in German. To ensure data privacy, physicians at the offices manually anonymized the discharge summaries. For this purpose, they received an online-training and were explicitly instructed to remove all direct personal identifiers, such as names and addresses, as well as quasi-identifiers, including surgery dates and hospital names. This was done in compliance with the General Data Protection Regulation and corresponded to the Health Insurance Portability and Accountability Act requirements for eliminating all 18 defined identifiers. Additionally, summaries containing information on rare diseases were excluded to enhance depersonalization.

To further ensure data protection, only the sections pertaining to diagnoses, epicrisis, medications, and recommendations were retained, as these were deemed most relevant for patient understanding. By excluding specific test results and other detailed information, we reduced the risk of re-identification through data aggregation. We obtained explicit informed consent from the participating physician offices to process the anonymized data by a third party for the purposes of this research. All anonymized discharge summaries processed by OpenAI’s ChatGPT were subjected to deletion protocols, ensuring they were deleted from OpenAI servers within 30 days following our deletion requests. Patient consent was not required, as the discharge summaries were de-identified by the treating physician. This approach was reviewed and deemed appropriate by the ethics committee of Witten/Herdecke University. While absolute anonymization cannot be guaranteed, the approach employed reduces the potential for re-identification, thereby ensuring a high standard of data privacy while preserving essential clinical information necessary for analysis.

### LLM simplification and augmentation with lifestyle recommendations

Between May 16th and May 21st, 2024, the 20 discharge summaries were inserted to ChatGPT via the online interface, utilizing the GPT-4o model (gpt-4o-2024-05-13) in its default configuration, with training data up to October 2023. The GPT-4o model was selected because of its strong performance in benchmark tests and its multilingual capabilities^22^.

We employed three different prompting approaches (see Supplementary Tables 1 and 2 for the specific prompts):

In the full-text simplification approach, each discharge summary was entered into the LLM in full, with a prompt to explain the content in simple language. This approach was taken to ensure that the model was provided with all necessary contextual information from each summary. This prompting approach employs zero-shot learning, in which the model provides an answer without being provided specific examples, and the chain-of-thought instruction to “proceed step by step” to enhance the model’s reasoning^23^. After each simplification, we started a new chat session.

In the segment-wise simplification approach, each of the four sections from original discharge summary were inserted separately into the LLM within a single chat session to leverage its in-context learning capabilities. This approach, akin to a divide-and-conquer strategy combined with few-shot prompting, enabled the model to reference its previous outputs while simplifying subsequent segments^24–26^. By doing so, the model effectively created a sequence of input-output examples or “shots” to learn from, which we hypothesized would enable it to process each segment more thoroughly, leading to more accurate and comprehensive simplifications.

For the lifestyle recommendation approach, the entire discharge summary was entered, and the model was prompted to generate personalized lifestyle recommendations.

These approaches resulted in 40 simplifications (20 from the full-text approach and 20 from the segment-wise approach) and 20 sets of lifestyle recommendations.

### Readability analysis

We evaluated the readability of the original discharge summaries and the LLM-generated simplifications using established readability metrics for the German-language. These included the Flesch-Reading-Ease formula as modified by Amstad (FRE-Amstad)^27^, the first Vienna Non-fiction Text Formula (WSTF)^28^, and the Lesbarkeitsindex (LIX)^29,30^. These metrics enable a quantitative and objective comparison of text readability.

The FRE-Amstad formula assigns readability scores on a scale from zero (most difficult) to 100 (easiest). The WSTF categorizes texts according to suitable school grade levels, with scores ranging from four (easiest) to 15 (most difficult), and the LIX formula scores range from 20 (easiest) to over 70 (most difficult). There are no specific readability guidelines for discharge summaries in Germany, as they are intended for communication among healthcare professionals. Thus, we compared the readability scores to the American Medical Association’s recommendation that health information for patients be written at or below a 6th-grade level (ages 11–12)^31^. For the FRE-Amstad, this readability target corresponds to a score of approximately 63, for the WSTF a score of six, and for the LIX a score of approximately 38^28^.

All readability metrics were computed using the software “TextLab” developed by H&H Communication Lab GmbH. Scores outside the predefined scale ranges for FRE-Amstad and WSTF were adjusted to the nearest valid scale boundary.

### Evaluation by medical experts

**Fig. 2:**
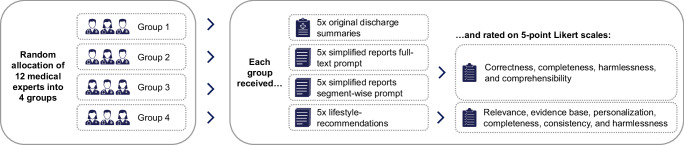
Workflow of the expert’s evaluation.

A convenience sample of 12 medical experts was recruited to evaluate the LLM-generated simplifications and lifestyle recommendations. Participation was limited to resident or specialist physicians in the fields of internal medicine or cardiology. To distribute the evaluation workload efficiently, the experts were divided into four groups of three. Each group was informed that an LLM had produced the content based on real-world discharge summaries. The evaluation process began with each expert reviewing an original discharge summary alongside one of its two simplified versions. This was followed by a structured questionnaire designed to assess the quality of the simplification. Subsequently, each expert assessed the second simplified version and then the lifestyle recommendations. This procedure was replicated across five distinct discharge summaries per group, ensuring each LLM-output was evaluated by three experts independently, resulting in 180 total ratings (Fig. 2).

The quality assessment categories were informed by prior research^14,16,32–35^ and included the following: For the simplified versions, the experts assessed correctness, completeness, harmlessness, and likely comprehensibility from a patient perspective using a 5-point Likert scale (1 = Strongly disagree; 5 = Strongly agree). For the lifestyle recommendations, the experts evaluated relevance, evidence base, personalization, completeness, consistency, and harmlessness, also using a 5-point Likert scale (1 = Strongly disagree; 5 = Strongly agree).

See Supplementary Table 3 for the specific survey statements and Supplementary Table 4 for an overview of the quality categories used in previous studies. We collected additional qualitative comments for each quality category through free-text responses. Additionally, we asked the experts a series of exploratory questions to better understand the current state of discharge reports and their willingness to use LLMs in clinical practice (see Supplementary Tables 6,7 and 8 for the results).

### Content analysis of LLM-generated lifestyle recommendations

To analyze the types and frequencies of LLM-generated lifestyle recommendations, we systematically examined all recommendations and developed categories that ensured an exhaustive and exclusive classification. Category development followed an iterative process based on Mayring’s inductive content analysis framework^36^. Each of the 20 recommendations was first reviewed to identify distinct individual suggestions, which were then grouped into broader recommendation categories through iterative refinement. Two authors (P.R. and J.F.) independently coded the data during the initial iteration, achieving a Cohen’s Kappa of 0.98, indicating near-perfect inter-rater agreement^37^. Discrepancies were resolved through consultation with a third author (L.F.). The software MAXQDA 2024 was utilized for both coding and analysis^38^.

### Statistical analysis

Differences in word count between the original discharge summaries and the LLM-simplifications were analyzed using Wilcoxon signed-rank tests. Statistical parameters for the responses on the Likert scales included median, 25%-quantile (Q1), 75%-quantile (Q3), interquartile range [IQR], minimum (Min), maximum (Max), mean, and standard deviation (*SD*). The intraclass correlation coefficient for the medical expert ratings was not computed because the number of discharge summaries analyzed was below the recommended minimum threshold of 30 samples^39^. Differences in readability scores between the original discharge summaries and the LLM-generated simplifications as well as the differences in the quality categories between the prompt approaches were analyzed using Wilcoxon signed-rank tests and adjusted for multiple testing using the Holm-Bonferroni method. A two-tailed test with *p* < 0.05 was considered statistically significant. All statistical analyses were performed using R software (version R-4.4.1).

### Reporting summary

Further information on research design is available in the Nature Portfolio Reporting Summary linked to this article.

## Results

The 20 discharge summaries analyzed showed considerable variation in documentation style, content, and length. These summaries originated from nine different hospitals, including five general hospitals, two specialty hospitals, and two university hospitals. Each hospital employed its own discharge documentation template. In total, the summaries covered nine different primary diagnoses and an average of three secondary diagnoses per summary (*SD* = 1.7). The average word count across all summaries was 268.4 (*SD* = 93.0). Detailed characteristics of the discharge summaries are provided in Supplementary Table 5.

The 12 recruited physicians had a median experience of 5.3 years [3-8.3], for detailed characteristics see Table 1.

**Table 1:** Characteristics of the medical experts. IQR = interquartile range

Characteristic	Values (*N* = 12)
**Age, median [IQR], years**	31.5 [30–36]
**Sex**	
Female	7 (58%)
Male	5 (42%)
**Specialty**	
Internal medicine	5 (42%)
Cardiology	7 (58%)
**Seniority**	
Resident	6 (50%)
Specialist	6 (50%)
**Work experience, median [IQR], years**	5.3 [3–8.3]

### Readability

**Fig. 3:**
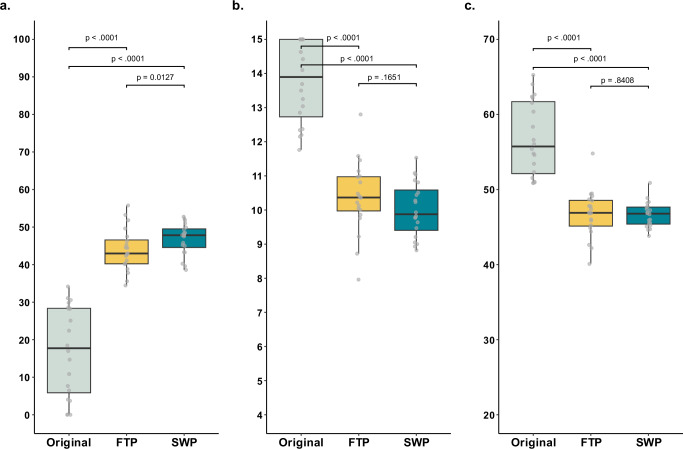
Differences in readability between original discharge summaries and LLM-simplifications using the full-text prompt (FTP) and segment-wise prompt (SWP). **a**–**c** Boxplots showing the comparison in readability metrics between the *N* = 20 original discharge summaries (gray) and their LLM simplifications using the full-text prompt (FTP, yellow) and segment-wise prompt (SWP, blue) approaches. **a** Scores for the modified Flesch-Reading-Ease (FRE-Amstad), with higher scores indicating better readability. **b** Scores for the Vienna Non-fiction Text Formula (WSTF), with lower scores indicating better readability. **c** Scores for Lesbarkeitsindex (LIX), with lower scores indicating better readability. The box spans the interquartile range, from the lower (25th) to the upper (75th) quartile, with the median marked by a line inside. Whiskers extend 1.5 times the interquartile range from the box edges. Statistical significance was assessed using nonparametric Wilcoxon signed-rank tests, with *p*-values adjusted for multiple comparisons using Holm-Bonferroni method.

The original discharge summaries exhibited poor readability, with a median FRE-Amstad of 17.7 [5.9-28.4], a median WSTF of 13.9 [12.7-15.0], and a median LIX of 55.7 [52.1-61.7] (Fig. 3). The LLM-simplified summaries generated with the full-text prompt were significantly more readable: FRE-Amstad increased from 17.7 to 43.0 (*p* < 0.0001), WSTF decreased from 13.9 to 10.4 (*p* < 0.0001), and LIX decreased from 55.7 to 46.9 (*p* < 0.0001). The segment-wise prompt improved FRE-Amstad from 17.7 to 47.8 (*p* < 0.0001), WSTF from 13.9 to 9.9 (*p* < 0.0001), and LIX from 55.7 to 46.8 (*p* < 0.0001). Despite significant readability improvements from both prompting approaches, the readability scores still exceeded the 6th-grade target level. When comparing the two prompting approaches, the segment-wise prompt yielded better readability scores than the full-text prompt, with the difference being statistically significant for the FRE-Amstad metric (*p* = 0.0127). Although the LLM-simplified summaries were more readable, they were significantly longer than the original versions. The original discharge summaries had a median word count of 287.0. In contrast, the LLM-simplified summaries using the full-text prompt had a median word count of 381.5 (*p* = 0.0005). The summaries generated using the segment-wise prompt contained nearly three times as many words compared to the originals (median word count = 866.0, *p* < 0.0001).

### LLM-generated simplified summaries

**Fig. 4:**
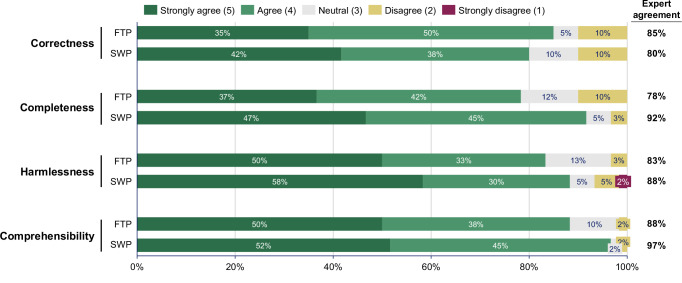
Quality assessment of the LLM-generated simplifications using the full-text prompt (FTP) and segment-wise prompt (SWP). Likert scale analysis of experts’ ratings of the LLM simplifications using the full-text prompt (FTP) and segment-wise prompt (SWP) approach. A total of *N* = 60 expert ratings were collected for the LLM-simplifications using FTP, and *N* = 60 ratings for the LLM simplifications using SWP. Expert agreement = Sum of all “agreed” and “strongly agreed” ratings. Percentages shown in the stacked bar chart may not sum to 100% due to rounding.

The 120 independent ratings by the 12 medical experts of the LLM-generated simplified discharge summaries using both prompting approaches covered correctness, completeness, harmlessness, and comprehensibility (Fig. 4 and Table 2). The medical experts agreed or strongly agreed that the simplifications generated with the full-text prompt were correct in 85% (51/60), complete in 78% (47/60), harmless in 83% (50/60), and comprehensible for a patient audience in 88% (53/60) of ratings. For the segment-wise prompt, the medical experts agreed or strongly agreed that the simplifications were correct in 80% (48/60), complete in 92% (55/60), harmless in 88% (53/60), and comprehensible for a patient audience in 97% (58/60) of ratings. The segment-wise prompt demonstrated a noticeable improvement in completeness, nearing statistical significance (*p* = 0.055). The segment-wise prompt also achieved higher ratings in harmlessness and comprehensibility than the full-text prompt (median = 5 [4-5] vs 4.5 [4-5] and median = 5 [4-5] vs. 4.5 [4-5], respectively), however, these differences were not statistically significant.

**Table 2:** Summary statistics for the quality assessment of the LLM-generated simplifications using the full-text- and segment-wise prompting approach

Prompt	Category	Median	Q1	Q3	IQR	Mean	*SD*	Min	Max
**Full-text**	Correctness	4.0	4.0	5.0	1.0	4.1	0.9	2.0	5.0
	Completeness	4.0	4.0	5.0	1.0	4.1	0.9	2.0	5.0
	Harmlessness	4.5	4.0	5.0	1.0	4.3	0.8	2.0	5.0
	Comprehensibility	4.5	4.0	5.0	1.0	4.4	0.7	2.0	5.0
**Segment-wise**	Correctness	4.0	4.0	5.0	1.0	4.1	1.0	2.0	5.0
	Completeness	4.0	4.0	5.0	1.0	4.4	0.7	2.0	5.0
	Harmlessness	5.0	4.0	5.0	1.0	4.4	0.9	1.0	5.0
	Comprehensibility	5.0	4.0	5.0	1.0	4.5	0.6	2.0	5.0

*N* = 60 expert ratings were collected for the LLM-simplifications using the full-text prompt, and *N* = 60 ratings for the LLM simplifications using the segment-wise prompt. 1 = Strongly disagree, 2 = Disagree, 3 = Neutral, 4 = Agree, 5 = Strongly agree. *IQR*  interquartile range.

Analysis of the experts’ free-text comments revealed recurring concerns about the potential harmfulness of LLM-simplifications, particularly incorrect or misleading information, the insensitive communication of findings that could lead to psychological distress, and the omission of medication information such as time of intake or dosage (Table 3). Expert 7 highlighted an example of inaccurate information where the LLM mistook a previous diagnosis for the current diagnosis: “*2 vessels WERE blocked, not ARE blocked. That makes a huge difference*”. An example of insensitive communication occurred when the LLM described “patent foramen ovale” as “You have a small hole in your heart that has been there since birth.” Expert 12 noted that this phrasing could sound alarming to the patient, even though the condition is generally harmless.

**Table 3:** Summary of medical expert’s comments regarding potential harmfulness

Category	Explanation	Frequency of mentions
**Inaccurate information**	The information provided is incorrect or misleading, such as the inclusion of assumptions not supported by the original discharge summaries.	7
**Insensitive communication**	Medical conditions or findings are described in a manner, such as using harsh language, inadequate explanations, or easily misunderstood terms, that could cause unnecessary alarm or distress to the patient.	7
**Medication issues**	Medication information is inaccurately described or incomplete, such as incorrect naming of drugs or omission of dosage details.	6
**Incomplete information**	Essential details are omitted from the summary, potentially leading to misunderstandings or risks to the patient if the information is not fully conveyed, especially in follow-up care or further medical procedures.	3
**Risk of self-treatment**	The information is presented in a way that could encourage the patient to engage in self-treatment.	2

Despite the high ratings for comprehensibility, experts highlighted instances where oversimplification was problematic: “[using] *‘A different heartbeat’ as a synonym for atrial flutter seems a little too superficial*’ (Expert 11). Additionally, the experts mentioned that the length of the summaries might pose a challenge for patients. However, they acknowledged that providing explanations, rather than simply translating medical jargon, could improve patient understanding: “[…] *the technical terms have been translated, but I’m not sure whether a patient without any background knowledge will be able to understand a mere translation*” (Expert 1).

### LLM-generated lifestyle recommendations

The original discharge summaries included a total of 32 lifestyle recommendations, 11 of which were unique. 15 recommendations focused on attending specific follow-up appointments or adhering to regular check-ups and screenings. Three discharge summaries had no lifestyle recommendations at all.

**Fig. 5:**
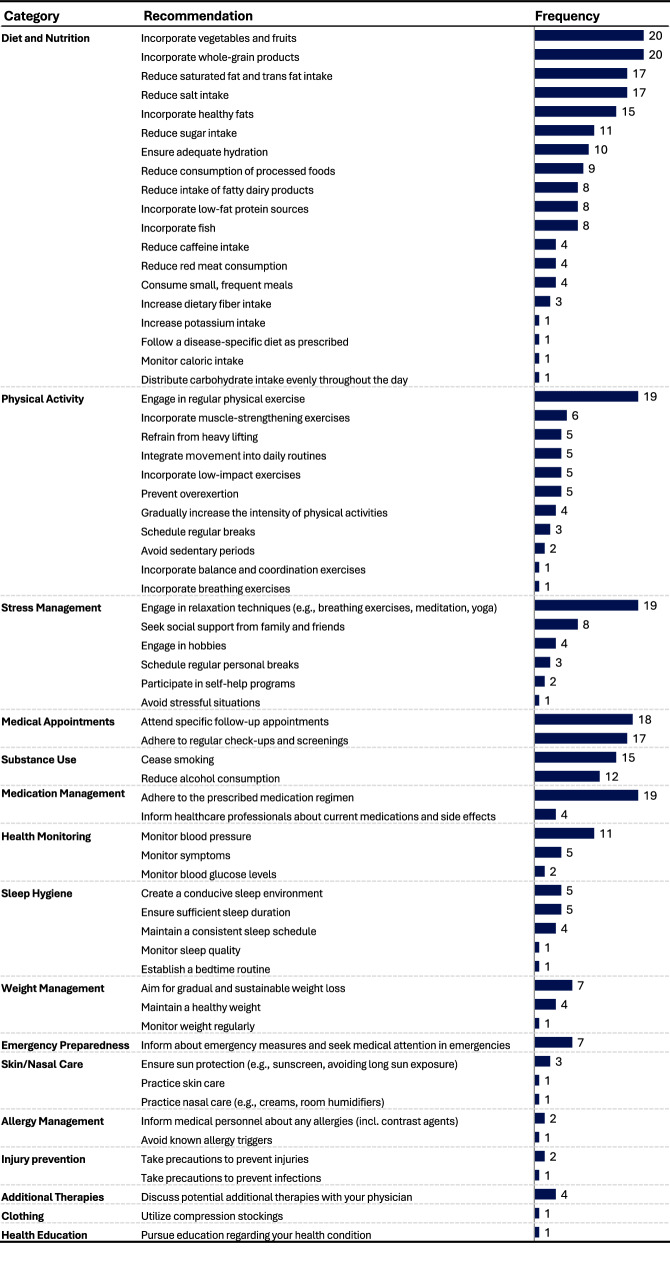
Overview of the LLM-generated lifestyle recommendations. Analysis of the frequency of the lifestyle recommendations across the *N* = 20 recommendation sets generated by the LLM.

For each of the 20 discharge summaries, the LLM generated a set of lifestyle recommendations (see Supplementary Fig. 1 for an example output of a recommendation set). Across all 20 sets, the model produced 410 recommendations, 64 of which were unique. These recommendations were categorized into 16 groups (Fig. 5). Recommendations addressing diet, physical activity, stress management, medical appointments, substance use, and medication management appeared in all 20 sets. The most frequently recommended actions, present in 18 or more of the 20 lifestyle recommendation sets, comprised consuming a diet rich in vegetables, fruits, and whole grains, engaging in regular physical exercise, practicing relaxation techniques, adhering to prescribed medication regimens, and attending specific follow-up appointments.

**Fig. 6:**
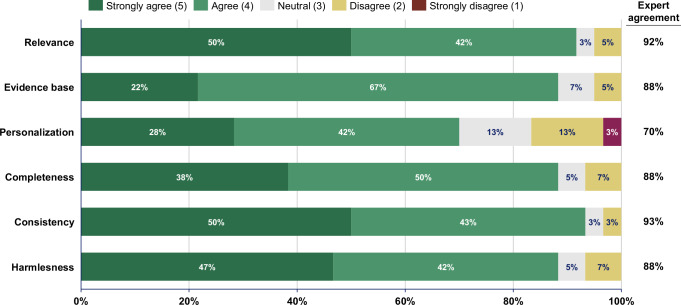
Quality assessment of the LLM-generated lifestyle recommendations. Likert scale analysis of experts’ ratings of the LLM-generated lifestyle recommendations. The 20 LLM-generated sets of lifestyle recommendations were rated by three experts resulting in *N* = 60 total ratings. Expert agreement = Sum of all “agreed” and “strongly agreed” ratings. Percentages shown in the stacked bar chart may not sum to 100% due to rounding.

The medical experts agreed or strongly agreed that the recommendations were relevant, evidence-based, complete, consistent, and harmless, in over 85% of ratings (Fig. 6 and Table 4). However, only 70% (42/60) of the ratings indicated agreement or strong agreement that the recommendations were personalized to individual patient conditions. The lack of personalization was also highlighted in the free-text comments, particularly concerning the need for sport activity recommendations to account for individual patient characteristics such as age or physical abilities (“*The recommendations are hardly personalized at all. For example, the question is whether the sports recommendation is suitable for a Parkinson’s patient*”, Expert 9). Experts generally agreed that the recommendations were evidence-based, though some noted that most recommendations should be classified as “Class C” and are not yet included in current clinical guidelines; for instance, one expert noted that “*Colchicine was proven in studies but not yet recommended in guidelines*” (Expert 12).

**Table 4:** Summary statistics for the quality assessment of the LLM-generated lifestyle recommendations

Prompt	Category	Median	Q1	Q3	IQR	Mean	*SD*	Min	Max
**Lifestyle-recommendations**	Relevance	4.5	4.0	5.0	1.0	4.4	0.8	2.0	5.0
	Evidence base	4.0	4.0	4.0	0.0	4.1	0.7	2.0	5.0
	Personalization	4.0	3.0	5.0	2.0	3.8	1.1	1.0	5.0
	Completeness	4.0	4.0	5.0	1.0	4.2	0.8	2.0	5.0
	Consistency	4.5	4.0	5.0	1.0	4.4	0.7	2.0	5.0
	Harmlessness	4.0	4.0	5.0	1.0	4.3	0.8	2.0	5.0

*N* = 60 expert ratings were collected for the LLM-generated lifestyle recommendations. 1 = Strongly disagree, 2 = Disagree, 3 = Neutral, 4 = Agree, 5 = Strongly agree. IQR = interquartile range.

## Discussion

To address the first objective of our study, we assessed the effectiveness of an LLM in improving the readability of hospital discharge summaries. Our findings show that LLMs can significantly enhance readability by reducing the required reading level from a college level to a 10th-grade level. While this marks a significant improvement, the reading level remains above the recommended 6th-grade level. Previous studies in radiology have demonstrated that LLMs can simplify text to below an 8th-grade reading level—the average reading level of a U.S. adult^34,40^. However, the original reports in those studies had a better baseline readability than the discharge summaries analyzed in this study, likely because radiology reports generally use a more concise and standardized format. We hypothesize that the effectiveness of LLM-simplifications is relative to the baseline complexity of the text. When the original content is highly complex, as with discharge summaries used in our study, improving readability is challenging and may result in a trade-off, in which simplification leads to a loss of critical information.

Notably, the LLM-generated outputs exhibited significantly improved readability and were highly rated by experts for their comprehensibility to medical laypeople, even though they contained a higher word count. The experts in our study also highlighted the importance of providing explanations rather than simply translating medical jargon, as this approach is more likely to improve patient understanding. Effective explanations require an understanding of the context and LLMs appear to have a distinct advantage in this regard, as they can dynamically capture complex contexts to guide their responses, unlike deterministic rule-based systems that, e.g., only provide layman translations of ICD-10 codes.

The adaptability and flexibility of LLMs do not come without risks. Although most simplifications generated by the LLM in our study were evaluated as correct, complete, harmless, and comprehensible, we identified several instances where the model produced potentially harmful inaccuracies. One of the primary concerns identified in our study is the generation of inaccurate information, known as “hallucinations” in the context of LLMs. Hallucinations occur when the LLM produces plausible-sounding but factually incorrect content^41^. For example, we found that prior diagnoses were mistakenly presented as current ones. This issue is likely due to the anonymization process used in our study, in which excluding diagnosis dates may have caused the model to confuse past and present medical conditions. Yet, such a misrepresentation of a patient’s medical history, along with other instances of incorrect information generated by the LLM, can have serious consequences in real-world settings, such as inappropriate treatment decisions and unnecessary anxiety for patients.

Another problem identified was instances of insensitive communication, which can lead to patient confusion and psychological distress. This underscores the need for communication that is both accurate and empathetic in patient-centered summaries. This presents an additional challenge for the LLM to adjust from the typically neutral style used in provider-to-provider communication to a more compassionate approach suitable for patients.

Perhaps the most critical issue was the recurring omission of medication dosage information. The absence of this information poses a great risk, especially if patients were to rely solely on these simplified summaries without consulting their healthcare providers. Interestingly, in our study, the omission of dosage information occurred only when using a full-text prompt. Similarly, transitioning from the full-text to the segment-wise prompt improved the output in terms of harmlessness, completeness, and comprehensibility. Although our study lacked the statistical power to detect significant differences, these improvements suggest that refining prompt design could mitigate some of the existing limitations of LLMs without modifying the underlying model architecture^15,35^. In addition, incorporating further mitigation strategies, such as a human-in-the-loop approach, appears indispensable for balancing the inherent strengths of LLMs with the potential errors stemming from their probabilistic nature—two sides of the same coin.

Overall, our findings regarding our first research objective align with previous research, demonstrating the potential of using LLMs in medical text simplification^34^. Our results also resonate with the assertion of Jeblick et al. that the primary goal of simplification should be to enhance clarity and comprehension rather than merely reducing text length^14^. We also concur with the growing consensus that implementing complementary safeguards and refraining from using LLMs as standalone solutions are essential to mitigate the significant errors they may produce^14,15,42^.

In addressing our second research objective, we evaluated the LLM’s capability to automatically generate lifestyle recommendations from discharge summaries. Our findings indicate that the LLM produced a substantial number of diverse lifestyle recommendations that medical experts generally consider to be relevant, evidence-based, complete, consistent, and harmless. This supports and extends previous research by demonstrating that LLMs can produce pertinent recommendations directly from real-world discharge summaries, without requiring specific queries^18^.

The integration of these recommendations could potentially complement pharmacological treatments and support primary, secondary and tertiary prevention, with minimal additional burden on the treating physician. However, a notable limitation is the generic nature of the recommendations produced. For instance, while advising cardiovascular exercise may be appropriate in many contexts, a patient with a foot injury would require a more tailored recommendation, such as low-impact exercises like swimming, to accommodate their clinical limitations. While some lack of personalization in our study may be partially attributable to the segmented and anonymized nature of the original discharge summaries, our observation of the generic nature of the LLM-generated recommendations aligns with previous research. A prior study demonstrated that, although LLMs can generate relevant and accurate treatment advice and lifestyle recommendations based on MRI reports, they still lack personalization^43^.

Several limitations of our study warrant acknowledgment. First, the relatively small sample size of 20 discharge summaries may limit the generalizability of our findings. Second, the exclusion of rare disease diagnoses may further restrict generalizability, as these conditions often require specialized discharge documentation. Third, the non-deterministic nature of LLM responses, characterized by variable outputs even with identical inputs, may affect the reproducibility of our results, as each prompt was applied exactly once per discharge summary. Fourth, our analysis was confined to a single LLM and did not include comparisons with domain-specific or fine-tuned models, which could potentially produce different outcomes. Fifth, while we employed standardized metrics to assess readability and gathered expert evaluations of comprehensibility from a patient’s perspective, these measures do not conclusively demonstrate that the texts are indeed easier for patients to understand. Sixth, due to the emerging nature of research on LLMs for medical text simplification, there is a lack of validated instruments designed to measure the quality of their outputs. Although we based the quality dimensions and Likert-scale assessments on previous studies to ensure comparability, there remains an urgent need for the development and validation of specific scales tailored to this application. Furthermore, the scope of this research precluded a systematic expert grading of the lifestyle recommendations or their comparison against clinical guidelines, which could have provided an additional dimension for quality assessment. Despite these limitations, this study is, to our knowledge, the first to apply the GPT-4o model to real-world cardiological discharge summaries to generate simple language explanations and lifestyle recommendations for German-speaking populations.

Future research should aim to address these limitations by incorporating a larger and more diverse sample of discharge summaries to enhance generalizability. Comparative analyses that explore different prompting techniques and assess both open-source and proprietary models would provide further insight into the performance and reliability of LLMs in clinical settings. It is also essential to directly engage patients in evaluating both objective comprehension and subjective satisfaction with the simplified texts, as well as to determine whether these modifications lead to improved health outcomes post-discharge. Furthermore, future studies should consider to systematically evaluate accountability, equity, security, fairness, and transparency in the design and deployment of these models, given the documented biases present in training data^44^. Unchecked biases risk producing unequal outcomes for different patient groups and may exacerbate existing health disparities. Finally, the development and validation of specialized instruments for assessing the quality of LLM outputs remain urgent priorities to ensure that such tools meet clinical and ethical standards in real-world healthcare environments.

Despite these efforts, broader concerns may persist around integrating LLMs into standard clinical workflows. As our study demonstrates, LLMs can generate misleading or harmful information, potentially compromising patient safety and raising ethical and legal questions about liability and malpractice. Additionally, the absence of a consensus on acceptable quality benchmarks and the limited research on real-world clinical accuracy complicate their acceptance by healthcare professionals. Concerns over data privacy pose another hurdle as entering personal data into LLMs without prior anonymization risks patient confidentiality^45^. While manual anonymization may be feasible in a research setting, it is impractical in a clinical environment and would negate the efficiency gains offered by LLMs’ automatic text generation. Potential solutions, such as employing anonymization algorithms, could be explored to ensure data privacy is maintained without compromising the functionality and utility of LLMs. Finally, LLMs, including GPT-4o, are not currently approved as medical devices and therefore cannot be used in clinical practice. However, the rapid and unregulated use of these models suggest that regulatory bodies will soon need to evaluate them. Such evaluations will present their own set of LLM-specific challenges^46,47^. Historically, the introduction of machine learning-based medical devices also faced regulatory hurdles. Nonetheless, as of December 2024, the U.S. Food and Drug Administration has authorized 1016 artificial intelligence-enabled medical devices^48^. We anticipate that, with further research and technological advancements, LLMs will eventually reach a risk/benefit threshold that allows them to obtain regulatory approval.

A promising development in this area is the emergence of open-source models. As highlighted by Riedemann, Labonne, and Gilbert, open-source models offer the most viable path to regulatory approval as medical devices^49^: Compared to closed-source models, open-source LLMs enable greater control over the model architecture, the source of training data, and update processes. Additionally, open-source models can help address data privacy concerns by allowing more stringent control over data flows, access rights, and enabling on-premise deployment. While there still appears to be a performance gap between open-source and closed-source LLMs, research has shown that fine-tuning open-source models for specific tasks can effectively close this gap^50^. These advantages make open-source models a compelling option for advancing their use in medical applications.

In conclusion, this study provides preliminary evidence that, with further development, LLMs could support the automated generation of patient-centered discharge summaries by improving readability while maintaining a reasonable, though imperfect, level of quality. While the LLM-generated lifestyle recommendations were generally of high quality, they lacked personalization, which may limit their clinical utility. Significant challenges remain, particularly concerning quality assurance, regulatory compliance, and data privacy. Further research is necessary to evaluate the real-world applicability, effectiveness, and safety of LLMs before they can be adopted in routine clinical practice.

## Supplementary information

Supplementary Material

Description of Additional Supplementary Materials

Supplementary Data 1

Supplementary Data 2

Supplementary Data 3

Reporting Summary

## Data Availability

The source data for Fig. 3 is available as Data 1, the source data for Fig. 4 is available as Data 2, the source data for Fig. 6 is available as Data 3. The original discharge summaries and simplified summaries generated by GPT-4o cannot be shared to ensure the data privacy of the patients. The anonymized expert survey responses are available from the corresponding author on reasonable request.
