# Supplementary material for: Evaluation of a large language model to simplify discharge summaries and provide cardiological lifestyle recommendations
Source: Commun Med (Lond). 2025 May 29;5:208. doi: 10.1038/s43856-025-00927-2 (PMC12122782; doi:10.1038/s43856-025-00927-2)
Supplement: Supplementary file 1 — Supplementary Material [file 43856_2025_927_MOESM1_ESM.pdf]

Supplementary Material

Supplementary Figure 1. Example of lifestyle recommendations generated by the large language model from one of the discharge summaries (translated from German).

Supplementary Table 1. Prompts used to generate simplified discharge summaries.

Supplementary Table 2. Prompt used to generate lifestyle recommendations.

Supplementary Table 3. Survey statements for assessing the quality of the simplifications and lifestyle recommendations generated by the large language model.

Supplementary Table 4. Quality categories used in previous studies for assessing the quality of simplifications generated by large language models.

Supplementary Table 5. Overview of original discharge summaries including primary diagnoses, number of secondary diagnoses, type of hospitals, readability and word count.

Supplementary Table 6. Exploratory questions to assess medical experts' opinions on patients' interest in understanding discharge reports, the comprehensibility of current discharge reports and the importance of including lifestyle recommendations.

Supplementary Table 7. Exploratory questions to assess medical experts' opinions on the prevalence of lifestyle recommendations in current discharge reports and patients' interest in these recommendations.

Supplementary Table 8. Exploratory questions to assess the medical experts' behavioral intentions regarding the use of large language models in clinical practice.

Supplementary References

Supplementary Figure 1. Example of lifestyle recommendations generated by the large language model from one of the discharge summaries (translated from German).

1. Blood Pressure Control
Since you suffer from high blood pressure and have already experienced a hypertensive crisis, it is important to regularly monitor and stabilize your blood pressure.

- **Blood Pressure Diary:** Measure your blood pressure daily in the morning and evening at the same time. Record the readings in a blood pressure diary.
- **Medication:** Take your Ramipril tablet every day at 8:00 AM. Do not discontinue the medication without consulting your doctor first.
- **Salt Reduction:** Reduce your salt intake. Use herbs and spices instead of salt to season your food.
- **Regular Check-ups:** Visit your doctor regularly to check your blood pressure and overall health.

2. Healthy Diet
A balanced diet can help lower your blood pressure and keep your heart healthy.

- **Fruits and Vegetables:** Eat at least five servings of fruits and vegetables daily.
- **Whole Grains:** Choose whole grain products like whole grain bread, oatmeal, and brown rice.
- **Low-fat Dairy Products:** Prefer low-fat or fat-free dairy products.
- **Healthy Fats:** Use vegetable oils like olive oil and eat fatty fish like salmon and mackerel twice a week.
- **Reduce Sugar:** Avoid sugary drinks and sweets.

3. Regular Exercise
Regular physical activity strengthens the heart and improves blood pressure.

- **Daily Activity:** Walk for at least 30 minutes every day. Use the stairs instead of the elevator.
- **Sports:** Swimming, cycling, or light jogging are good for your heart. Try to exercise three times a week.
- **Heart-Friendly Exercises:** Practice exercises like yoga or Pilates, which are relaxing and can reduce stress.

4. Weight Control
Maintaining a healthy body weight relieves stress on your heart and helps regulate blood pressure.

- **Healthy Diet:** Follow the dietary recommendations mentioned above.
- **Regular Exercise:** Combine endurance training with muscle-strengthening exercises.
- **Target Weight:** Discuss a healthy target weight with your doctor and the best ways to achieve it.

5. Stress Management
Stress can increase blood pressure. It is important to develop strategies for managing stress.

- **Relaxation Techniques:** Regularly practice relaxation techniques such as meditation, breathing exercises, or progressive muscle relaxation.
- **Time for Yourself:** Take time each day for activities that bring you joy and relaxation.
- **Social Support:** Talk to family and friends about your feelings and seek support when you need it.

6. Smoking Cessation and Alcohol Consumption
Smoking and excessive alcohol consumption are risk factors for cardiovascular diseases.

- **Quit Smoking:** If you smoke, seek support to quit. There are various programs and tools that can help you.
- **Alcohol Consumption:** Drink alcohol in moderation. This means no more than one glass of wine or beer per day for women and two glasses for men.

7. Regular Medical Check-ups
Your health condition requires regular medical check-ups to ensure your treatment is optimal.

- **Doctor Visits:** Regularly visit your cardiologist and general practitioner to monitor your health.
- **Blood Tests:** Have regular blood tests to monitor your levels.

8. Skincare for Psoriasis
Psoriasis can be worsened by stress and certain triggers. Good skincare is important.

- **Moisturizing:** Use moisturizing creams or ointments daily.
- **Avoiding Triggers:** Identify and avoid triggers that may worsen your psoriasis, such as certain foods or stress.
- **Medication:** Take your prescribed psoriasis medications regularly and consult your dermatologist if needed.

Supplementary Table 1. Prompts used to generate simplified discharge summaries.

Prompt	**Original Wording** (German)	**Translation** (English)
**Full-text**	<Aufgabe><br>Du erhältst einen kardiologischen Arztbrief aus einem Krankenhaus. Bitte erkläre dem Patienten bzw. der Patientin den Arztbrief in laienverständlicher Sprache.<br><br>Halte dich dabei an folgende Anweisungen:<br>• Verzichte auf medizinische Fachwörter und verwende stattdessen allgemein verständlich Begriffe.<br>• Erkläre medizinische Konzepte und Zusammenhänge so ausführlich, dass ein Patient oder eine Patientin ohne medizinisches Wissen sie leicht nachvollziehen kann.<br>• Verwende einfaches Deutsch, das auch Personen mit geringer Lesekompetenz verstehen können.<br>• Richte deine Erklärung direkt an den Patienten oder die Patientin und verwende „Sie“.<br>• Verzichte auf eine Anrede oder Verabschiedung.<br>• Deine Antwort sollte ausführlich und gut strukturiert sein.<br><br>Gehe Schritt für Schritt vor.<br><Kardiologischer Arztbrief>	<Task><br>You will receive a cardiology medical report from a hospital. Please explain the report to the patient in layman's terms.<br><br>Follow these instructions:<br>• Avoid using medical jargon and instead use terms that are easy to understand.<br>• Explain medical concepts and connections in detail so that a patient without medical knowledge can easily follow them.<br>• Use simple language that can be understood by people with low literacy.<br>• Direct your explanation to the patient and use “You” (polite).<br>• Do not include a salutation or closing.<br>• Your response should be detailed and well-structured.<br><br>Proceed step by step.<br><Cardiology Medical Report>
**Segment-wise**	<Aufgabe><br>Du erhältst ein Abschnitt aus einem kardiologischen Arztbrief aus einem Krankenhaus. Bitte erkläre dem Patienten bzw. der Patientin den Abschnitt in laienverständlicher Sprache.<br><br>Halte dich dabei an folgende Anweisungen:<br><br>• Verzichte auf medizinische Fachwörter und verwende stattdessen allgemein verständlich Begriffe.<br>• Erkläre medizinische Konzepte und Zusammenhänge so ausführlich, dass ein Patient oder eine Patientin ohne medizinisches Wissen sie leicht nachvollziehen kann.<br>• Verwende einfaches Deutsch, das auch Personen mit geringer Lesekompetenz verstehen können.<br>• Richte deine Erklärung direkt an den Patienten oder die Patientin und verwende „Sie“.<br>• Verzichte auf eine Anrede oder Verabschiedung.<br>• Deine Antwort sollte ausführlich und gut strukturiert sein.<br><br>Gehe Schritt für Schritt vor.<br><Abschnitt Kardiologischer Arztbrief>	<Task><br>You receive a section from a cardiology medical report from a hospital. Please explain the section to the patient in layman's terms.<br><br>Follow these instructions:<br><br>• Avoid using medical jargon and instead use terms that are easy to understand.<br>• Explain medical concepts and connections in detail so that a patient without medical knowledge can easily understand them.<br>• Use simple language that can be understood by people with low reading skills.<br>• Direct your explanation to the patient and use “You” (polite).<br>• Do not include a salutation or closing.<br>• Your response should be detailed and well-structured.<br><br>Proceed step by step.<br><Section of the Cardiology Medical Report>

Supplementary Table 2. Prompt used to generate lifestyle recommendations.

<table>
<tr><th>Prompt</th><th>Original Wording (German)</th><th>Translation (English)</th></tr>
<tr><td>Recommen-dation</td><td><Aufgabe>
Erstelle maßgeschneiderte Lebensstilempfehlungen basierend auf den detaillierten Angaben des folgenden kardiologischen Arztbriefes.

Bitte berücksichtige dabei:

• Orientiere dich an den aktuellen Leitlinien und Empfehlungen renommierter medizinischer Fachgesellschaften
• Passe die Lebensstilempfehlungen spezifisch an den individuellen Gesundheitszustand und die Bedürfnisse des Patienten bzw. der Patientin an.
• Verwende einfaches Deutsch, das auch Personen mit geringer Lesekompetenz verstehen können.
• Formuliere die Empfehlungen detailliert, präzise und in einer für Laien verständlichen Sprache, um eine leichte Umsetzbarkeit zu gewährleisten.
• Richte deine Erklärung direkt an den Patienten oder die Patientin und verwende „Sie“.
• Verzichte auf eine Anrede oder Verabschiedung.
• Deine Antwort sollte ausführlich und gut strukturiert sein.

Gehe Schritt für Schritt vor.

<Kardiologischer Arztbrief></td><td><Task>
Create customized lifestyle recommendations based on the detailed information in the following cardiology medical report.

Please consider the following:

• Base your advice on the current guidelines and recommendations of reputable medical societies.
• Tailor the lifestyle recommendations specifically to the patient's individual health condition and needs.
• Use simple language that can be understood by people with low reading skills.
• Formulate the recommendations in a detailed, precise, and easy-to-understand manner to ensure they can be easily implemented.
• Direct your explanation to the patient and use “You” (polite).
• Do not include a salutation or closing.
• Your response should be detailed and well-structured.

Proceed step by step.

<Cardiology Medical Report></td></tr>
</table>

Supplementary Table 3. Survey statements for assessing the quality of the simplifications and lifestyle recommendations generated by the large language model

Output	Quality category	Question
**Simplification**	Correctness	The patient letter is a factually accurate translation of the original letter.
	Completeness	The patient letter includes all the relevant medical information from the original discharge letter necessary for the patient.
	Harmlessness	The patient letter contains no incorrect or misleading information that could lead to physical or psychological harm.
	Comprehensibility	The patient letter uses terms, expressions, and a language that are easily understood by the average patient.
**Lifestyle recommen-dation**	Relevance	The lifestyle recommendations are relevant for the patient.
	Evidence base	The lifestyle recommendations follow current clinical guidelines and/or are based on medically established practices.
	Personalization	The lifestyle recommendations are personalized and take the patient's specific state of health into account as to the extent apparent.
	Completeness	The lifestyle recommendations cover all lifestyle aspects that are necessary for the prevention, treatment or management of the patient's specific disease.
	Consistency	The lifestyle recommendations are consistent with each other.
	Harmlessness	The lifestyle recommendations contain no incorrect or misleading information that could lead to physical or psychological harm.

Note. All statements were translated from German and assessed using a 5-point Likert scale (1 = Strongly disagree; 5 = Strongly agree).

Supplementary Table 4. Quality categories used in previous studies for assessing the quality of simplifications generated by large language models.

Study	Field	Quality measurements
Ali et *al.*[1]	Dermatology	*Assessed by medical professionals*:<br>• Factual correctness (10-point Likert scale)<br>• Humanness (10-point Likert scale)
Schmidt et *al.*[2]	Radiology	*Assessed by medical professionals*:<br>• Factual correctness (5-point Likert scale)<br>• Presence of relevant medical information (5-point Likert scale)<br>• Simplicity and understandability (5-point Likert scale)<br>• Potentially incorrect conclusions or harmfulness (5-point Likert scale)<br>*Assessed by non-medical personnel:*<br>• Simplicity and understandability of words (5-point Likert scale)<br>• Simplicity and understandability of sentences (5-point Likert scale)<br>• High density of information (5-point Likert scale)<br>• Knowing what the text is about (5-point Likert scale)<br>• Wishing that the text was clearer about importance (5-point Likert scale)<br>• Ability to draw the right conclusions (5-point Likert scale)<br>• Text informed as well as a healthcare professional (5-point Likert scale)<br>• Full understanding of what the injury or illness was (5-point Likert scale)
Jeblick et *al.*[3]	Radiology	*Assessed by medical professionals*:<br>• Factual correctness (5-point Likert scale)<br>• Completeness (5-point Likert scale)<br>• Potential harm (5-point Likert scale)
Chung et *al.*[4]	Radiology/ Oncology	*Assessed by medical professionals*:<br>• Factual correctness (5-point Likert scale)<br>• Understanding (5-point Likert scale)<br>• Completeness (5-point Likert scale)<br>• Potential for harm (5-point Likert scale)<br>• Overall quality (5-point Likert scale)<br>• Likelihood to send to patient (5-point Likert scale)
Salam et *al.*[5]	Radiology/ Cardiology	*Assessed by medical professionals*:<br>• Factual correctness (5-point Likert scale)<br>• Completeness (5-point Likert scale)<br>• Serious misinformation with potential harm (5-point Likert scale)<br>*Assessed by non-medical personnel:*<br>• Understandability (5-point Likert scale)
Kim et *al.*[6]	Cardiology/ Surgery	*Assessed by doctors or nurses:*<br>• Factuality (5-point Likert scale)<br>• Comprehensiveness (5-point Likert scale)<br>• Usability<br>*Assessed by non-medical personnel:*<br>• Ease (5-point Likert scale)<br>• Fluency (5-point Likert scale)

Supplementary Table 5. Overview of original discharge summaries including primary diagnoses, number of secondary diagnoses, type of hospitals, readability and word count.

Letter	Primary diagnosis	No. of secondary diagnoses	Hospital	Type of hospital	FRE-Amstad	LIX	WSTF	Word count
1	Pulmonary embolism	2	1	General	30.6	50.9	12.3	181
2	Heart failure	5	2	Specialty	4.0	62.2	15.0	266
3	Acute myocardial infarction	3	3	University	10.9	56.6	14.6	154
4	Chronic ischemic heart disease	4	3	University	17.0	56.1	14.1	380
5	Medical observation and evaluation for suspected diseases	3	3	University	3.7	62.7	15.0	326
6	Chronic ischemic heart disease	4	3	University	18.5	54.8	13.5	378
7	Rheumatic mitral valve diseases	7	3	University	14.7	58.4	14.4	318
8	Chronic ischemic heart disease	5	4	General	28.2	52.3	12.9	243
9	Transplanted organ and tissue status	2	5	Specialty	34.2	50.9	11.8	327
10	Chronic ischemic heart disease	0	3	University	6.5	61.5	15.0	126
11	Atrial fibrillation and flutter	1	6	General	0.0	64.0	15.0	95
12	Occlusion and stenosis of precerebral arteries	3	6	General	22.4	55.4	13.7	190
13	Chronic ischemic heart disease	2	3	University	25.1	50.9	12.4	336
14	Chronic ischemic heart disease	5	1	General	28.6	51.5	12.2	234
15	Chronic ischemic heart disease	4	7	General	28.3	54.6	13.3	367
16	Heart failure	4	3	University	0.0	62.4	15.0	341
17	Chronic ischemic heart disease	1	8	University	29.8	53.4	13.0	408
18	Heart failure	3	2	Specialty	0.1	65.3	15.0	186
19	Atrial fibrillation and flutter	4	9	General	31.1	51.0	12.2	308
20	Atrial fibrillation and flutter	1	6	General	7.7	60.4	15.0	204

Note. For the modified Flesch-Reading-Ease (FRE-Amstad), higher scores indicate better readability. For the Vienna Non-fiction Text Formula (WSTF) and the Lesbarkeitsindex (LIX), lower scores reflect better readability.

Supplementary Table 6. Exploratory questions to assess medical experts' opinions on patients' interest in understanding discharge reports, the comprehensibility of current discharge reports and the importance of including lifestyle recommendations.

Question	Median	Q1	Q3	IQR	Mean	*SD*	Min	Max
How would you assess the average interest of patients in understanding their discharge letter?	4.0	3.0	4.0	1.0	3.9	0.7	3.0	5.0
How would you assess the comprehensibility of average discharge letters from the perspective of patients?	2.0	1.0	2.0	1.0	1.6	0.6	1.0	3.0
How would you assess the importance of integrating lifestyle recommendations into discharge letters for patients?	4.0	3.0	5.0	2.0	4.0	1.1	2.0	5.0

Note. *N* = 11 experts responded to these questions using a 5-point Likert scale (1 = Very low; 5 = Very high). The questions were translated from German. Q1 = 25%-quantile; Q3 = 75%-quantile; IQR = interquartile range; Min = minimum; Max = maximum; *SD* = standard deviation.

Supplementary Table 7. Exploratory questions to assess medical experts' opinions on the prevalence of lifestyle recommendations in current discharge reports and patients' interest in these recommendations

Question	Median	Q1	Q3	IQR	Mean	*SD*	Min	Max
How high do you estimate the proportion (0-100%) of discharge letters that contain lifestyle recommendations in relation to all cardiology discharge letters? *(0% = No discharge letter contains lifestyle recommendations;100% = All discharge letters contain lifestyle recommendations)*	24.0	20.0	52.5	32.5	34.2	21.6 pp	10.0	70.0
How high do you estimate the proportion (0-100%) of patients who express interest in lifestyle recommendations compared to the total number of patients? *(0% = No patient is interested; 100% = All patients are interested)*	60.0	48.3	69.8	21.5	55.8	20.9 pp	10.0	80.0

Note. *N* = 12 experts responded to these questions using a sliding scale with endpoints at 0% and 100% (in 1% increments). The questions were translated from German. Q1 = 25%-quantile; Q3 = 75%-quantile; IQR = interquartile range; Min = minimum; Max = maximum; *SD* = standard deviation; pp = percentage points.

Supplementary Table 8. Exploratory questions to assess the medical experts' behavioral intentions regarding the use of large language models in clinical practice

Question	Median	Q1	Q3	IQR	Mean	SD	Min	Max
If a large language model is used to generate simplified patient letters in my hospital/clinic/practice,...								
...I would intend to use the large language model.	5.0	4.8	5.0	0.3	4.7	0.6	3.0	5.0
...I would use the large language model.	5.0	4.8	5.0	0.3	4.7	0.6	3.0	5.0
...I plan to use the large language model.	5.0	4.0	5.0	1.0	4.6	0.6	3.0	5.0

Note. N = 12 experts responded to these questions using a 5-point Likert scale (1 = Strongly disagree; 5 = Strongly agree). The questions were based on the measurement of behavioral intentions from the Unified Theory of Acceptance and Use of Technology 2[7] and were translated from German. Q1 = 25%-quantile; Q3 = 75%-quantile; IQR = interquartile range; Min = minimum; Max = maximum; SD = standard deviation.

References

1. Ali, S. R., Dobbs, T. D., Hutchings, H. A. & Whitaker, I. S. Using ChatGPT to write patient clinic letters. *The Lancet. Digital health* **5,** e179-e181; 10.1016/S2589-7500(23)00048-1 (2023).

2. Schmidt, S., Zimmerer, A., Cucos, T., Feucht, M. & Navas, L. Simplifying radiologic reports with natural language processing: a novel approach using ChatGPT in enhancing patient understanding of MRI results. *Arch Orthop Trauma Surg* **144,** 611–618; 10.1007/s00402-023-05113-4 (2024).

3. Jeblick, K. *et al.* ChatGPT makes medicine easy to swallow: an exploratory case study on simplified radiology reports. *European radiology* **34,** 2817–2825; 10.1007/s00330-023-10213-1 (2024).

4. Chung, E. M. *et al.* Feasibility and acceptability of ChatGPT generated radiology report summaries for cancer patients. *Digital health* **9,** 20552076231221620; 10.1177/20552076231221620 (2023).

5. Salam, B. *et al.* Generative Pre-trained Transformer 4 makes cardiovascular magnetic resonance reports easy to understand. *Journal of cardiovascular magnetic resonance : official journal of the Society for Cardiovascular Magnetic Resonance* **26,** 101035; 10.1016/j.jocmr.2024.101035 (2024).

6. Kim, H., Jin, H. M., Jung, Y. B. & You, S. C. Patient-Friendly Discharge Summaries in Korea Based on ChatGPT: Software Development and Validation. *Journal of Korean medical science* **39,** e148; 10.3346/jkms.2024.39.e148 (2024).

7. Venkatesh, Thong & Xu. Consumer Acceptance and Use of Information Technology: Extending the Unified Theory of Acceptance and Use of Technology. *MIS Quarterly* **36,** 157; 10.2307/41410412 (2012).
