# Supplementary material for: Evaluation of a large language model to simplify discharge summaries and provide cardiological lifestyle recommendations
Source: Commun Med (Lond). 2025 May 29;5:208. doi: 10.1038/s43856-025-00927-2 (PMC12122782; doi:10.1038/s43856-025-00927-2)
Supplement: Supplementary file 2 — Description of Additional Supplementary Materials [file 43856_2025_927_MOESM2_ESM.pdf]

Description of Additional Supplementary Files

File name: Supplementary Data 1

Description: The source data for Fig. 3

File name: Supplementary Data 2

Description: the source data for Fig. 4

File name: Supplementary Data 3

Description: the source data for Fig. 6
