# Supplementary material for: Evaluation of a large language model to simplify discharge summaries and provide cardiological lifestyle recommendations
Source: Commun Med (Lond). 2025 May 29;5:208. doi: 10.1038/s43856-025-00927-2 (PMC12122782; doi:10.1038/s43856-025-00927-2)
Supplement: Supplementary file 6 — Reporting Summary [file 43856_2025_927_MOESM6_ESM.pdf]

Corresponding author(s): Leonard Fehring

Last updated by author(s): Apr 12, 2025

Reporting Summary

Nature Portfolio wishes to improve the reproducibility of the work that we publish. This form provides structure for consistency and transparency in reporting. For further information on Nature Portfolio policies, see our Editorial Policies and the Editorial Policy Checklist.

Statistics

For all statistical analyses, confirm that the following items are present in the figure legend, table legend, main text, or Methods section.

n/a	Confirmed	
☐	☒	The exact sample size (*n*) for each experimental group/condition, given as a discrete number and unit of measurement
☐	☒	A statement on whether measurements were taken from distinct samples or whether the same sample was measured repeatedly
☐	☒	The statistical test(s) used AND whether they are one- or two-sided<br>*Only common tests should be described solely by name; describe more complex techniques in the Methods section.*
☒	☐	A description of all covariates tested
☐	☒	A description of any assumptions or corrections, such as tests of normality and adjustment for multiple comparisons
☐	☒	A full description of the statistical parameters including central tendency (e.g. means) or other basic estimates (e.g. regression coefficient) AND variation (e.g. standard deviation) or associated estimates of uncertainty (e.g. confidence intervals)
☒	☐	For null hypothesis testing, the test statistic (e.g. *F*, *t*, *r*) with confidence intervals, effect sizes, degrees of freedom and *P* value noted<br>*Give P values as exact values whenever suitable.*
☒	☐	For Bayesian analysis, information on the choice of priors and Markov chain Monte Carlo settings
☒	☐	For hierarchical and complex designs, identification of the appropriate level for tests and full reporting of outcomes
☒	☐	Estimates of effect sizes (e.g. Cohen's *d*, Pearson's *r*), indicating how they were calculated

Our web collection on statistics for biologists contains articles on many of the points above.

Software and code

Policy information about availability of computer code

Data collection	We used the online survey platform LimeSurvey (6.1.8 (build 230717) to collect the expert evaluations. All data was stored in MS Excel.
Data analysis	All statistical analyses were performed using R software (version R-4.4.1). The software MAXQDA 2024 was utilized for coding the lifestyle recommendations. All readability metrics were computed using the software “Textlab” developed by H&H Communication Lab GmbH.

For manuscripts utilizing custom algorithms or software that are central to the research but not yet described in published literature, software must be made available to editors and reviewers. We strongly encourage code deposition in a community repository (e.g. GitHub). See the Nature Portfolio guidelines for submitting code & software for further information.

Data

Policy information about availability of data

All manuscripts must include a data availability statement. This statement should provide the following information, where applicable:

- Accession codes, unique identifiers, or web links for publicly available datasets
- A description of any restrictions on data availability
- For clinical datasets or third party data, please ensure that the statement adheres to our policy

The original discharge summaries and simplified summaries generated by GPT-4o cannot be shared to ensure the data privacy of the patients. The anonymized expert survey responses and the dataset used for readability analysis in this study are available from the corresponding author on reasonable request.

Research involving human participants, their data, or biological material

Policy information about studies with human participants or human data. See also policy information about sex, gender (identity/presentation), and sexual orientation and race, ethnicity and racism.

Reporting on sex and gender	We collected information on the sex of the participating physicians. This information was not used for further analysis.
Reporting on race, ethnicity, or other socially relevant groupings	N/A
Population characteristics	Age, sex medical specialty (internal medicine, cardiology), seniority (resident, specialist) and work experience in years
Recruitment	A convenience sample of 12 medical experts was recruited to evaluate the LLM-generated simplifications and lifestyle recommendations. Participation was limited to resident or specialist physicians in the fields of internal medicine or cardiology.
Ethics oversight	University of Witten/Herdecke

Note that full information on the approval of the study protocol must also be provided in the manuscript.

Field-specific reporting

Please select the one below that is the best fit for your research. If you are not sure, read the appropriate sections before making your selection.

☐ Life sciences ☒ Behavioural & social sciences ☐ Ecological, evolutionary & environmental sciences

For a reference copy of the document with all sections, see nature.com/documents/nr-reporting-summary-flat.pdf

Behavioural & social sciences study design

All studies must disclose on these points even when the disclosure is negative.

Study description	We conducted a prospective exploratory study using real-world, anonymized discharge summaries of patients with cardiological conditions. These summaries were processed through a large language model (GPT-4o) to simplify the content and incorporate personalized lifestyle recommendations. The generated outputs were evaluated for readability using software tools and assessed for quality by a panel of medical experts.
Research sample	- 20 anonymized hospital discharge summaries from two German medical practices<br>- 12 medical experts (resident or specialist physicians in the fields of internal medicine or cardiology)
Sampling strategy	Convenience sample; Inclusion criteria: Resident or specialist physicians in the fields of internal medicine or cardiology
Data collection	The anonymized hospital discharge reports were scanned and digitized into text documents. As part of an online survey, the medical experts reviewed both the original discharge reports and the simplified versions, providing their assessments. The experts were informed that the summaries were produced by a large language model (non-blinded) but were unaware of which of the two distinct prompting approaches was used to generate each version (blinded).
Timing	March 2024 - July 2024
Data exclusions	No data was excluded
Non-participation	Three participants declined participation.
Randomization	Random allocation of the experts into four groups

Reporting for specific materials, systems and methods

We require information from authors about some types of materials, experimental systems and methods used in many studies. Here, indicate whether each material, system or method listed is relevant to your study. If you are not sure if a list item applies to your research, read the appropriate section before selecting a response.

Materials & experimental systems

n/a	Involved in the study
☒	☐ Antibodies
☒	☐ Eukaryotic cell lines
☒	☐ Palaeontology and archaeology
☒	☐ Animals and other organisms
☒	☐ Clinical data
☒	☐ Dual use research of concern
☒	☐ Plants

Methods

n/a	Involved in the study
☒	☐ ChIP-seq
☒	☐ Flow cytometry
☒	☐ MRI-based neuroimaging

Plants

Seed stocks	*Report on the source of all seed stocks or other plant material used. If applicable, state the seed stock centre and catalogue number. If plant specimens were collected from the field, describe the collection location, date and sampling procedures.*
Novel plant genotypes	*Describe the methods by which all novel plant genotypes were produced. This includes those generated by transgenic approaches, gene editing, chemical/radiation-based mutagenesis and hybridization. For transgenic lines, describe the transformation method, the number of independent lines analyzed and the generation upon which experiments were performed. For gene-edited lines, describe the editor used, the endogenous sequence targeted for editing, the targeting guide RNA sequence (if applicable) and how the editor was applied.*
Authentication	*Describe any authentication procedures for each seed stock used or novel genotype generated. Describe any experiments used to assess the effect of a mutation and, where applicable, how potential secondary effects (e.g. second site T-DNA insertions, mosiacism, off-target gene editing) were examined.*
